# Lung perfusion: MRI vs. SPECT for screening in suspected chronic thromboembolic pulmonary hypertension

**DOI:** 10.1002/jmri.25714

**Published:** 2017-04-04

**Authors:** Christopher S. Johns, Andrew J. Swift, Smitha Rajaram, Paul J.C. Hughes, David J. Capener, David G. Kiely, James M. Wild

**Affiliations:** ^1^ Academic Unit of Radiology University of Sheffield, Royal Hallamshire Hospital Sheffield South Yorkshire UK; ^2^ Pulmonary Vascular Institute, Royal Hallamshire Hospital Sheffield South Yorkshire UK

**Keywords:** chronic thromboembolic pulmonary hypertension, pulmonary hypertension, dynamic contrast enhanced, perfusion, magnetic resonance imaging

## Abstract

**Purpose:**

To assess the diagnostic accuracy of magnetic resonance imaging (MRI) perfusion against perfusion single photon emission tomography (SPECT) screening for chronic thromboembolic pulmonary hypertension (CTEPH). Ventilation/perfusion (V/Q) scintigraphy is recommended to screen for suspected CTEPH. It has previously been shown that 3D dynamic contrast‐enhanced (DCE) lung perfusion MRI has a similar sensitivity for diagnosing CTEPH in comparison to planar perfusion scintigraphy; however, planar scintigraphy has now been largely replaced by SPECT, due to higher spatial resolution and sensitivity.

**Materials and Methods:**

Consecutive patients with suspected CTEPH or unexplained pulmonary hypertension attending a referral center, who underwent lung DCE perfusion MRI at 1.5T, perfusion SPECT, and computed tomography pulmonary angiography (CTPA) within 14 days of right heart catheterization, from April 2013 to April 2014, were included. DCE‐MR, SPECT, and CTPA were independently analyzed by two blinded radiologists. Disagreements were corrected by consensus. The gold standard reference for the diagnosis of chronic thromboemboli was based on a review of multimodality imaging and clinical findings.

**Results:**

In all, 74 patients with suspected CTEPH underwent all three modalities. Forty‐six were diagnosed with CTEPH (36) or chronic thromboembolic disease (CTED) (10). 3D DCE perfusion MRI correctly identified all patients (sensitivity of 100%), compared with a 97% sensitivity for SPECT.

**Conclusion:**

DCE lung perfusion MRI has increased sensitivity when compared with perfusion scintigraphy in screening for CTEPH. As MRI does not use ionizing radiation, it should be considered as a first‐line imaging modality in suspected CTEPH.

**Level of Evidence:** 3

**Technical Efficacy:** Stage 3

J. Magn. Reson. Imaging 2017;46:1693–1697.

Chronic thromboembolic pulmonary hypertension (CTEPH) is a potentially curable form of pulmonary hypertension (PH).^1^ The diagnosis requires a mean pulmonary artery pressure (mPAP) ≥25 mmHg at right heart catheterization (RHC), in the presence of at least one segmental defect on perfusion imaging or filling defects on computed tomography pulmonary angiography (CTPA), after at least 3 months of effective anticoagulation.^2^ The true incidence and prevalence of CTEPH is not known, but the cumulative incidence of CTEPH after survival from an acute pulmonary embolus is reported as 3.8% at 2 years.^3^ The pathological process is thought to be due to incomplete lysis of the acute pulmonary embolus; the subsequent organization of the obstructing thrombus leading to obstruction of pulmonary vascular bed.^4^ This ultimately leads to increased pulmonary arterial pressure, right ventricular dysfunction, and if untreated the prognosis is poor.^5^

Patients with CTEPH usually have a history of either pulmonary embolism or deep venous thrombosis, although a significant proportion may present with unexplained breathlessness or pulmonary hypertension of unknown cause.^6^, ^7^ It is important that the diagnosis of CTEPH is made, as pulmonary endarterectomy is associated with increased survival and a favorable functional outcome in CTEPH.^1^ The 2013 World Symposium on Pulmonary Hypertension recommended single photon emission computed tomography (SPECT) ventilation/perfusion (V/Q) scintigraphy as the preferred screening test for CTEPH,^8^ but this entails injection of 100 MBq of 99mTc‐labeled macroaggregated human albumin, resulting in exposure to ionizing radiation with an effective dose of 0.017 mSv/MBq.^9^

Cardiopulmonary magnetic resonance imaging (MRI) is emerging as an important tool for assessing the structure and function of the right ventricle in patients with PH,^10^ and it has already been shown that 3D dynamic contrast‐enhanced (DCE) lung perfusion MRI has a similar sensitivity for diagnosing CTEPH when compared with planar perfusion scintigraphy.^11^ Planar scintigraphy is increasingly being replaced by SPECT in clinical practice, due to the higher spatial resolution and improved sensitivity in the detection of smaller perfusion defects.^12^ Thus, the aim of this study was to assess the diagnostic accuracy of MRI perfusion against perfusion SPECT as a screening tool for CTEPH.

## Patients and Methods

Consecutive patients with suspected CTEPH or unexplained pulmonary hypertension attending a pulmonary hypertension referral center^13^ who underwent contrast‐enhanced lung perfusion MRI, perfusion SPECT, and CTPA within 14 days of right heart catheterization, from April 2013 to April 2014, were identified. A diagnosis of CTEPH was based on a review of multimodality imaging, clinical correlates, and right heart catheterization as per standard clinical criteria^2^; this was decided at a multi‐disciplinary team meeting and was used as the reference standard. Patients with chronic thromboembolic disease, but without pulmonary hypertension, were considered a true positive. This was decided since the current method of diagnosis of pulmonary hypertension is not made on imaging, but instead relies on pressure measurements in the pulmonary artery on right heart catheterization. The local Research Ethics Committee granted ethical approval for this retrospective study, and written consent was waived.

### Image Acquisition

MRI was performed on a 1.5T whole body system (HDx, GE Healthcare, Milwaukee, WI) using a time‐resolved 3D spoiled gradient echo sequence with view‐sharing. An 8‐channel cardiac receiver array coil was used. The sequence parameters were: TE = 1.1 msec, TR = 2.5 msec, flip angle 30°, field of view 48 × 48 cm, parallel imaging in plane ×2, in‐plane resolution 200 × 80, bandwidth 250 kHz, slice thickness 10 mm, ∼32 slices, 48 timepoints with an overall effective 3D frame rate of ∼0.5 seconds. Images were acquired in a coronal orientation during a single breath‐hold. The acquired voxel size was 1.875 × 1.875 × 10 mm. Contrast injection of a 0.05 ml per kg patient weight dose of Gd‐BT‐D30A (Gadovist, Schering, Berlin, Germany) was injected at a rate of 4 ml per second with the injection rate controlled using an activated pump injector (Spectris, MedRad, Pittsburgh, PA) typically via a vein in the antecubital fossa using an 18G cannula, followed by a 20‐ml saline flush.

SPECT imaging was performed on a GE Infinia SPECT system using a low energy general‐purpose collimator; 100 MBq 99mTc MAA was administered through a direct intravenous injection with a needle of 21G or larger. The image acquisition parameters were: acquisition matrix 128 × 128, 60 projections per detector and 7 seconds per projection. Images were acquired prone with the patient's arms extended above their heads, where possible.

### Image Analysis

**Figure 1:**
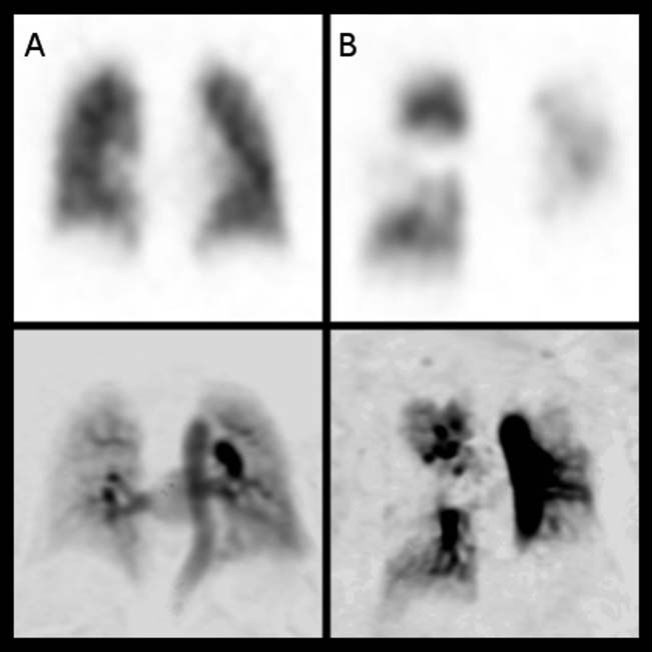
Matched slices from 3D coronal SPECT perfusion images (top row) and DCE MR perfusion images (bottom) in a patient with normal lung perfusion **(A)** and with CTEPH **(B)**. This shows the typical wedge‐shaped perfusion defects (arrows) in the right mid, left lower, and left upper zones on the MR and the SPECT imaging of patient B. Note the images are presented on an inverse gray scale as reviewed clinically.

DCE perfusion images were analyzed on a slice‐by‐slice basis by subtraction of the baseline precontrast image; this was performed on a GE Advantage workstation. The peak enhancement image in the contrast passage time series was independently analyzed by a general radiologist (C.S.J., 5 years of experience) and a consultant chest radiologist (A.J.S., 11 years of experience) blinded to all other imaging and clinical information. The images were reviewed on a general reporting workstation in the general radiology department on diagnostic quality Barco screens (Barco, UK). The images were qualitatively assessed as either positive or negative for chronic thromboembolic disease. On both DCE perfusion MRI and perfusion SPECT, the presence of one or more segmental or subsegmental perfusion defects was considered positive for pulmonary embolic disease, as per recognized clinical guidelines.^14^ Figure 1 gives an example of a normal and positive SPECT and DCE‐MRI scan. The DCE perfusion images were typically viewed with a window of 40 and a level of 19, although this was manipulated if required. Subsequently, the SPECT imaging was reviewed by the same radiologists, at a separate sitting, separated from the time of the MRI analysis by at least 1 week, blinded to all other imaging and clinical information. Any disagreements were resolved by consensus. The multidisciplinary decision of the presence or absence of chronic thromboembolic disease, as outlined above, was considered the reference standard.

### Statistical Analysis

Diagnostic accuracy was assessed for SPECT and DCE perfusion MRI using a 2×2 predictive table to calculate sensitivity, specificity, negative and positive predictive value. Interobserver and intertest agreement was assessed using kappa, with 0.60–0.79 considered moderate agreement, 0.80–0.89 strong, and above 0.90 excellent agreement.^15^ Statistical analysis was performed using SPSS 22 (IBM, Chicago, IL) and GraphPad Prism 7 (GraphPad Software, San Diego, CA). *P* < 0.05 was considered statistically significant.

### Patient Demographics

Over the 1‐year period of the study, 74 patients with suspected CTEPH attended for perfusion MRI, SPECT, and CTPA. Thirty‐six patients were diagnosed with CTEPH and 10 patients with CTED (chronic thromboembolic disease without pulmonary hypertension) according to standard criteria. In the CTEPH and CTED groups there were 20 female and 26 male patients. The mean age of both groups was 62 years (standard deviation 14 years).

## Results

DCE perfusion MRI correctly identified all CTEPH and CTED patients (sensitivity of 100%), compared to 97% sensitivity for SPECT; *P*‐values for all data were < 0.0001 (see Table 1 for more details). The specificity of MR was 81% and SPECT 81%. The patient not identified by SPECT had mild, inoperable CTEPH, and was correctly identified on CTPA and perfusion MRI. There was one indeterminate SPECT case and two indeterminate MRI cases. The kappa value between SPECT and MRI was 0.88, indicating strong agreement. Interobserver kappa was 0.80 and 0.88 for SPECT and MRI, respectively, indicating strong interobserver agreement.

**Table 1:** Summary of Diagnostic Performance of SPECT and MR Perfusion

	SPECT perfusion	Perfusion MRI
Sensitivity	97% (95% CI 88–99%)	100% (95% CI 92–100%)
Specificity	81% (95% CI 62–94%)	81% (95% CI 62–94%)
Positive predictive value	90% (95% CI 78–97%)	90% (95% CI 78–97%)
Negative predictive value	96% (95% CI 78–100%)	100% (95% CI 85–100%)
Interobserver agreement (kappa)	0.80	0.88

The *P*‐values for all data were < 0.0001.

## Discussion

DCE lung perfusion MRI has increased sensitivity when compared to SPECT perfusion scintigraphy in the detection of CTEPH. In the patients studied, a combination of perfusion MR and CTPA identified all patients with CTEPH and CTED. This reflects and updates the findings of a previous study by Rajaram et al that compared DCE perfusion MRI with planar scintigraphy.^11^ There were two indeterminate sets of MRI scans; it was felt that low signal‐to‐noise ratios in these images was the underlying reason for an indeterminate study. Perfusion MRI can be performed in the same sitting as high‐resolution pulmonary MR angiography and cardiac MRI scan, and has the potential for a “one‐stop‐shop” analysis of pulmonary perfusion and assessment of right heart and pulmonary vascular characteristics. Cardiac MR assessment of baseline and progression of right ventricular characteristics over time in idiopathic pulmonary artery hypertension (IPAH) has been previously shown to be a predictor of outcome.^16^, ^17^

These results differ somewhat from the literature regarding MR in the diagnosis of acute pulmonary emboli (PE). The PIOPED III study assessed the efficacy of MR angiography (MRA), and showed a sensitivity of 78% for acute PE detection in technically adequate scans; 25% of patients had technically inadequate scans.^18^ Although it should be noted that, while contrast‐enhanced MRA and DCE perfusion images are different methods for assessment of the pulmonary vasculature, in that MRA focuses on structural form of the major vessels while DCE perfusion MR highlights downstream perfusion of the small vessels, it is likely that the main difference in the sensitivity of MR in the assessment of acute and chronic PE could be due to the size of thrombus detected. Due to improvements in technologies, modern‐day CTPA is able to pick up very small subsegmental acute PEs, which (in our opinion), are likely to be smaller than those that can be currently detected on MRA or MR perfusion imaging. These peripheral acute emboli are very unlikely to cause CTEPH, so the lower spatial resolution of MRA and perfusion imaging when compared to CTPA should not miss clinically significant chronic thromboembolic disease. In the present study, DCE‐MRI had the highest sensitivity for detecting CTEPH, although SPECT Q only failed to identify a single patient with distal thromboembolic disease. Importantly, however, no modality missed surgically accessible CTEPH. The case that was missed by SPECT was in a patient with coexistent lung pathology, which caused a defect that was not typical of embolic disease on the SPECT image; the anatomical information available on the nonsubtracted MRI database meant that was less of an issue for the MR perfusion scan. It should be noted that CTEPH and CTED may be missed on CTPA by radiologists not experienced in the assessment of pulmonary vascular disease, leading to the recommendation in the latest international guidelines that SPECT Q is preferred to CTPA when screening for CTEPH.^14^ Given the similarities between the images obtained by DCE‐MRI and SPECT Q, it is anticipated that DCE‐MRI would have similar diagnostic performance in the hands of a general radiologist.

Although SPECT imaging and DCE perfusion MRI both demonstrate pulmonary perfusion of the small (subvoxel size) vessels, there are fundamental differences in the method of acquisition and contrast enhancement. SPECT imaging represents deposition of radio‐isotope particles in the capillaries and small arterioles in the lung,^9^ with acquisition times around 10 minutes in a pseudosteady‐state of lung perfusion. DCE MR perfusion images are, however, dynamically acquired in the first pass of gadolinium; and a 3D dataset is acquired (here, approximately every 0.5 sec) during a breath‐hold. The initial “unenhanced” prebolus arrival dataset is subtracted from the peak enhancement dataset to give the perfusion images. As such, the MR images are interpreted as a snapshot of the “peak” first‐pass perfusion signal and also the enhanced signal from blood in the conducting major vessels is not explicitly segmented from the signals from the rest of the pulmonary blood pool.

**Figure 2:**
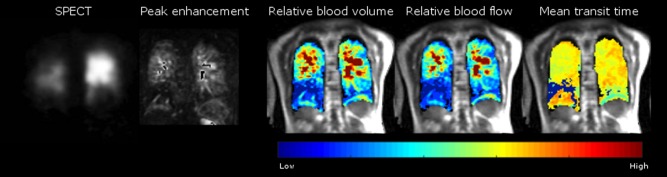
Matched slices from a single patient with CTEPH showing the SPECT and the peak enhancement image from a DCE perfusion MRI scan used clinically, alongside semiquantitative perfusion maps (pulmonary blood volume, flow, and mean transit time) from a DCE perfusion MRI.

An alternative method of analysis that might closer represent the cumulative signal of a SPECT scan would be to integrate the dynamic perfusion signal with time to create maps of regional perfused blood volume.^19^ An example of the quantitative parametric maps of pulmonary perfusion is provided in Fig. 2. Using the arterial input function and unenhanced lung *T*_1_ maps, time‐contrast curves can be calculated for each voxel and peak contrast, mean transit time, and pulmonary perfusion can be calculated, as previously described.^20^, ^21^ These are calculated for each voxel over the time‐course of the perfusion dataset and can be presented in a parametric map. Techniques to segment out the major vessels could also be employed to mask the signal from the perfused capillary bed,^22^ although background signal from the major vessels was not felt to affect the radiological interpretation of the images in this study.

This study has a number of limitations. As our study was conducted in a pulmonary hypertension referral center,^13^ the negative and positive predictive value will only be valid for a population where the probability of CTEPH and CTED is high, although given the high sensitivity and specificity of MRI, it would be expected to perform well in symptomatic patients following PE where the prevalence of CTEPH is increased. The retrospective nature of the study has the potential to introduce bias; however, both reviewers were blinded to each other's observations and clinical information. A prospective study examining the value of MRI and SPECT VQ as a screening test for CTEPH will be required to address the clinical utility and diagnostic performance of these investigations in populations at risk.

In conclusion, MRI has high sensitivity for CTEPH and does not use ionizing radiation making it an ideal imaging screening test for patients with suspected CTEPH.
